# Per-Cooling (Using Cooling Systems during Physical Exercise) Enhances Physical and Cognitive Performances in Hot Environments. A Narrative Review

**DOI:** 10.3390/ijerph17031031

**Published:** 2020-02-06

**Authors:** Wafa Douzi, Olivier Dupuy, Dimitri Theurot, Juhani Smolander, Benoit Dugué

**Affiliations:** University of Poitiers, Laboratoire Mobilité Vieillissement Exercice (MOVE)-EA6314, Faculty of Sport Sciences, 8 Allée Jean Monnet, 86000 Poitiers, France; wafa.douzi01@univ-poitiers.fr (W.D.); olivier.dupuy@univ-poitiers.fr (O.D.); dimitri.theurot@univ-poitiers.fr (D.T.); smolanderjuhani@gmail.com (J.S.)

**Keywords:** “Aerobic” exercises, “anaerobic” exercises, cooling, cooling devices, hyperthermia, thermoregulation

## Abstract

There are many important sport events that are organized in environments with a very hot ambient temperature (Summer Olympics, FIFA World Cup, Tour de France, etc.) and in hot locations (e.g., Qatar). Additionally, in the context of global warming and heat wave periods, athletes are often subjected to hot ambient temperatures. It is known that exercising in the heat induces disturbances that may provoke premature fatigue and negatively affects overall performance in both endurance and high intensity exercises. Deterioration in several cognitive functions may also occur, and individuals may be at risk for heat illnesses. To train, perform, work and recover and in a safe and effective way, cooling strategies have been proposed and have been routinely applied before, during and after exercise. However, there is a limited understanding of the influences of per-cooling on performance, and it is the subject of the present review. This work examines the influences of per-cooling of different areas of the body on performance in terms of intense short-term exercises (“anaerobic” exercises), endurance exercises (“aerobic” exercises), and cognitive functioning and provides detailed strategies that can be applied when individuals train and/or perform in high ambient temperatures.

## 1. Introduction

Undertaking physical exercise in hot conditions increases one’s core temperature and may impair performance regardless of the intensity or the duration of exercise. There is a large amount of scientific literature concerning this topic in terms of maximal exercise (lasting 3–10 min) [1,2,3,4,5], isometric contractions (maximum voluntary contraction or sustained maximal isometric contractions), repeated sprints [6], prolonged exercises at intensities varying from 40 to 80% of maximal oxygen consumption (VO_2_ max) and lasting 1 h or more [7,8,9,10,11,12]. Moreover, physical exercise in a hot environment (ambient temperature over 30 °C) also negatively affects mental performance and cognitive function (i.e., attention, memory [13,14,15,16,17], and executive function [14,18,19]).

Thus, exercising in the heat induces thermoregulatory and other physiological strains that may impair performance capacity. To reduce this strain and optimize performance, some interventions can be adopted, such as acclimation (laboratory or other arranged situations) and acclimatization (natural environment) to heat [20,21]. However, to effectively perform acclimatization, athletes need to be careful with their hydrated state. It has been advised to drink 6 mL of fluid per kg of body mass every 2–3 h before starting to exercise [22] and to minimize dehydration when exercising [23,24].

In addition, in thermally challenging environments such as the Summer Olympics, athletes can implement strategies to enhance heat loss by using commercial cooling systems (e.g., cooling vests) and other cooling interventions (e.g., ice packs, ice ingestion, fans, cold-water immersion). In particular, temperatures are expected to exceed 40 °C in the 2022 FIFA World Cup (Qatar). Thus, the optimization of cooling strategies for physical performance has gained interest and has been the topic of several reviews [22,25] and is of continuing interest in basic and applied research [26,27].

Therefore, the effects of a variety of cooling methods that differ in duration, method and site of application on different kinds of performance have been investigated. Concerning the use of cooling during exercise (per-cooling), several tools have been developed that can be placed on different body areas and can be used during training and competitions in the heat. To date, there is only one meta-analysis (that we recently published) that synthesizes what is known on the impact of cooling different parts of the body on different kinds of physical performance, and the meta-analysis should be considered a companion work of the present narrative review. In the former meta-analysis, we were able to show that per-cooling improves both ‘aerobic’ and ‘anaerobic’ exercise performance with a greater benefit for ‘aerobic’ exercise and that the magnitude of the effect depends on the type and site of the cooling application [28].

The present work extends this former work and reviews what is known concerning (1) exercise-induced hyperthermia and performance decreases; (2) the effects of cooling during exercise on physical (‘aerobic’ and ‘anaerobic’ exercise) and cognitive performance; (3) the effects of a cooled body area on physical and cognitive performance; (4) the underlying physiological mechanisms of cooling and (5) practical considerations of per-cooling strategies and some perspectives on future research.

## 2. Exercise-Induced Hyperthermia and Performance Detriments

Hyperthermia is defined as an increase in one’s core temperature above 37 °C at rest and 38 °C during moderate-intensity exercise [29]. A hot environmental condition is an extra thermal load that induces a detrimental effect on exercise performance and capacity [30,31,32,33,34]. It has been shown that heat exposure induces the production of heat-shock proteins (HSPs), which are referred to as stress proteins [35] that function as a protective mechanism. However, heat exposure leads to other problems such as the stretching of muscle fibres, glycogen depletion, muscular ischaemia, and oxygen radical release that may induce oxidative stress [36].

### 2.1. Hyperthermia and Aerobic Performance

According to the study by Gastin [37], who discussed the contributions of energy systems (aerobic and anaerobic) during exercise, exercises of a duration of 76 s or longer are considered predominantly ‘aerobic’. Prolonged physical activity provokes homeostatic disturbances that may affect athletic exercise performance and lead to premature fatigue [38]. This phenomenon can be explained by challenging the limits of the human cardiovascular system and temperature regulation capabilities in a very hot condition [23,39]. For a long time, it has been suggested that heat strain impairs endurance performance (e.g., marching in the desert) [40], and this concept has been confirmed both in laboratory [31,41] and field settings [42].

There is a growing body of literature showing decreases in performance during submaximal exercise with hyperthermia. Several works have evaluated the impact of hyperthermia on time to exhaustion (TTE) tests (incremental or at a constant work rate), time trial (TT) tests (self-paced) and VO_2_ max tests [1,2,7,9,11,12,31,41,43,44,45,46,47].

Regardless of the manner of intensity adjustments (exercise protocols), TTE and TT studies demonstrated that hyperthermia impairs aerobic exercise performance. For instance, it has been shown that an increase in external temperature from 20 °C to 40 °C was sufficient to reduce TTE at a 70% VO_2_ peak from 67 ± 1 min (mean ± SEM) to 30 ± 3 min [48]. Other studies (e.g., [30]) also support this finding by comparing different ambient temperatures ranging from 4 to 31 °C; it has been shown that a higher ambient temperature corresponds to a shorter TTE performance (for instance, TTE was shortened by ∼42 min in the warmest studied environment (31 °C)). Similarly, in self-paced exercise (TTs), hyperthermia induced a degradation of aerobic performance by a reduction in power output [11,12]. For instance, Periard and colleagues [49] reported that TT (40 km) performance was degraded by ∼13% (in mean power output) in a hot-humid environment compared to a temperate environment. In line with these findings, elite cyclists who performed two TTs both in hot (32 °C) and temperate (23 °C) conditions presented a TT performance that was degraded by ∼6% in a hot-humid environment [47]. In addition, by examining the impact of graded heat stress on VO_2_ max, Arngrímsson and co-workers suggested that VO_2_ max decreases incrementally with step-wise increases in ambient temperature [1]. This finding is in agreement with older studies where a slight increase in ambient temperature induced a slight decrease in VO_2_ max (<3%) [50,51]. Such a reduction is not always observed since the slight increase in ambient temperature during a submaximal exercise may not be enough to decrease exercise performance [52,53,54]. However, following preheating using 20 min of low-intensity exercise at 46 °C, a marked decrease (27%) in VO_2_ max [4] was observed. Furthermore, a combined increase in core (39 °C) and skin temperature (37 °C) following preheating with a water-perfused system provoked a 16% decrease in VO_2_ max [9].

It is well recognized that an elevated core temperature in hot conditions during prolonged exercise may challenge human regulatory systems [22], resulting in changes such as cardiovascular strain [55,56,57], glycogen depletion [58], perceptual discomfort [59,60], and central nervous system dysfunction [3]. In turn, these disturbances may hinder endurance exercise performance in response to the development of hyperthermia [61].

**Summary:**

**i.**  Via the mechanisms cited above, hyperthermia impairs aerobic performance in general, including performance both in short time to exhaustion exercises and longer time trials.
**ii.**  It seems worth noting that environmental high temperature can challenge the limits of human regulatory systems, along with an elevated risk for hyperthermia. Prolonged exercise in the heat requires special attention from the coaching staff and athletes.

### 2.2. Hyperthermia and ‘Anaerobic’ Performance

According to the study by Gastin [37], exercises with a duration of 75 s or less are considered predominantly ‘anaerobic’. Regarding brief high-intensity exercise, it is difficult to draw a clear conclusion about the effect of environmental high temperature on performance; some studies depicted no changes [9,62,63], and others showed a decrease [3] or even an improvement [64,65] in performance.

During brief and intense exercises such as the single sprint (SS), an improvement in performance can be obtained following muscle heating [66,67,68,69]. With this method, Sargeant [70] reported that there was an increase in maximal peak force and power by ∼11% during a 20-s maximal cycling effort, while cooling the legs in an 18 or 12 °C water bath resulted in a reduction in maximal peak force by 12% and 21%, respectively. Similarly, Guy and co-workers [71] found better performance during 100-m and 200-m sprints in hot environments (∼2% faster performance) compared with cooler environmental conditions. The enhanced performance in brief intense exercise might be explained by the rate of phosphocreatine (PCr) utilization in hot conditions [66], and this improvement can also be explained by accelerations in the conduction velocity of muscle fibres with high muscle temperatures [66,72]. In contrast, other studies did not report any effects of hot ambient temperature on intense exercise performance. Performance in anaerobic exercise may not be affected by hot or humid conditions when athletes can correctly re-hydrate and are able to warm-up before anaerobic exercises [62,73,74].

Nonetheless, the beneficial effects of an elevated muscle temperature on sprint performance can be impaired in hot environments when the exercise is repeated (e.g., a repeated series of sprint exercises (RS)) [67]. It is known that individuals with hyperthermia (40 °C) demonstrate decreased performance during sustained contractions compared with individuals in temperate conditions (18 °C) [3,75]. Additionally, it has been reported [76] that the power output at the onset of a sprint is lower under hot and dry conditions (12.2 ± 1.5 W·kg^−1^) than under thermoneutral conditions (12.6 ± 1.4 W·kg^−1^) during repeated cycling sprints (RCS) with short recovery periods.

During repeated sprint exercises in the heat, performance can be compromised owing to rising levels of NH3 concentrations, which may disturb the cerebral neurotransmitter homeostasis and provoke central fatigue [77,78].

Hyperthermia slows down muscle blood flow and may jeopardize arterial oxygen delivery to the muscle being exercised, thereby leading to a greater reliance on anaerobic energy contributions. Thus, ATP and PCr levels decline with muscle lactate and H+ accumulation [79].

Undertaking short and intense physical exercise during hot environmental conditions compared with temperate conditions can disrupt some regulatory systems, owing to an accelerated decline in cardiac output, perfusion pressure and blood flow in the exercising muscles, leading to a more rapid decrease in oxygen delivery and oxygen uptake and provoking a higher level of fatigue [2,80].

**Summary:**

➢ In summary, although an increase in muscle temperature may improve power production during short-duration exercise, this beneficial effect disappears if the exercise is repeated and significant performance decrements occur with exacerbated hyperthermia.

### 2.3. Cognitive Performance in the Heat

Although the physiological responses to hyperthermia are well documented, the cognitive responses remain equivocal. Some studies reported a negative effect of hyperthermia on cognitive function [14,15,81,82], while others were not able to show any effects [83,84].

It has previously been shown that an environmental temperature greater than 29.4 °C impairs cognitive skills, tracking performance, and dual task performance [85]. Indeed, high temperature can impair various types of cognitive performance, such as attention [14,86], memory [14,15,16,17], vigilance [87] and executive function [14,18,19]. For instance, hyperthermia provoked deficits both in vigilance and multi-choice reaction time tasks [88] and elevated pilot errors [89]. However, it seems that hyperthermia primarily affects the most complex cognitive tasks [14].

Hot environmental conditions can exacerbate poor cognitive functioning, thereby degrading performance outcomes [90]. This phenomenon can be explained by the high level of cognitive function required for an optimal performance (i.e., decision making) [91].

It seems that a slight increase in core temperature improves cognitive performance [92]. This improvement may result from an increase in arousal and cerebral blood flow [93]. However, as highlighted by Bandelow et al. [94], when one’s core temperature reaches 37.8 °C, the improvement in cognitive performance disappears. Thus, the authors speculated that heat strain overloads one’s cognitive capacity when his or her core temperature exceeds this threshold [92]. More recently, Yeganeh et al., (2018) performed a meta-analysis and confirm that hyperthermia is accompagnied by a deterioration of cognitive performance and reported that the mean percentage decline in cognitive performance as the result of variation in temperature follows an inverted U-shape curve.

To understand the mechanisms responsible for altered cognitive function in individuals with hyperthermia, Nunneley and co-workers [95] used positron emission tomography (PET) to examine regional changes in the cerebral metabolic rate of humans with steady-state hyperthermia. Their results showed changes in activity of the lateral cerebellum in individuals with hyperthermia, and it was suggested that the altered function in this area impaired cognitive function. More generally, many neurophysiological disturbances can explain the decrease in cognitive performance during exercise in the heat [61]. Among these disturbances, one can note disturbances at the level of neurotransmission, but also a decrease in the oxygen supply observed by a reduction in cerebral blood flow and cerebral oxygenation. Therefore, performance deteriorates when the total amount of cognitive resources are insufficient for both completing a task and combating hyperthermia [14,96].

Also often associated with hyperthermia, water loss can cause brain disturbances and decrease cognitive performance [97,98,99]. However, the exact role of dehydration on cognitive functions need to be further clarified.

From a practical perspective, the changes in cognitive function cause risks and functional implications in hot environments owing to an alteration of working memory [96] or errors in judgement and decision making [100].

**Summary:**

➢ Hyperthermia (stressor) places an additional demand on the limited cognitive workspace, thereby degrading cognitive performance and leading to potential risks for athletes performing in hot conditions.

## 3. Effects of Cooling during Exercise (Per-Cooling)

Cooling prior to exercise (pre-cooling) has gained the most attention in research, and it has been inferred that pre-cooling enhances performance in subsequent activities (especially in ‘aerobic’ exercises) by lowering the level of thermal strain experienced during exercise [7,101]. However, the changes induced by pre-cooling, such as a significant vasoconstriction and/or decrease in muscular temperature [102], may impair sprint performance, especially when the active muscles are directly pre-cooled [103].

However, cooling during exercise has received considerably less research attention. This relative lack of interest in this area may be explained by issues regarding practicality (e.g., excess weight, skin irritation and sport regulations) [104]. Some recent studies suggested that the advantages gained from cooling during exercise may outweigh those of pre-cooling [105,106]. Cooling during exercise is named per-cooling (‘per’ is derived from the Latin word meaning ‘during’) [27]. Per-cooling has been used to minimize the negative effects of hyperthermia and to augment performance [107].

Per-cooling studies differ with respect to the cooling technique that is used, such as the use of a cooling garment [108,109], cold water immersion [110,111], ice slurry ingestion [112,113], cold fluid ingestion [114,115], cold packs [116,117,118,119,120], a cooling vest (10–20 °C) [121,122], an ice vest [123,124], a ventilated vest [125,126], a water spray [105], and air ventilation (fanning) [127,128]. Taken together, the majority of per-cooling techniques are effective in improving exercise performance in hot and temperate conditions, and it is advisable to use sufficient cooling power during exercise to reach an optimal effect. Hence, the effectiveness of the cooling intervention used during exercise is dependent on the volume of cooling and the extent of the thermal strain experienced [26].

Several reviews and meta-analyses have recently been published on the benefits of per-cooling [27,107,129]. However, no clear conclusion has been drawn regarding the effects of per-cooling on different types (i.e., ‘aerobic’, ‘anaerobic’ and cognitive) of performance.

### 3.1. Per-Cooling and Aerobic Performance

Despite the paucity of research in this area, it has been acknowledged that using cooling interventions during endurance exercises may improve exercise performance [27].

The per-cooling strategies that are beneficial in the heat include the following:

The ingestion of cold fluids and an ice slurry, both with [130,131] and without menthol,

The cooling of the neck and face region via a cooling collar, ice packs or water poured on the head and face [105,116,117,118,119,120,132],

The cooling of the torso via a cooling vest [121,122], ice vest [123,124], and ventilated vest [125,126],

The use of cooling garments [108,109,133] and hand cooling via a hand cooling device [134,135,136], and Air ventilation [128,137].

To assess the effects of per-cooling interventions during prolonged exercise, time to exhaustion tests have extensively been used [114,117,132]. It has been reported that continuous facial fanning during cycling exercises at 75% of maximal oxygen uptake significantly increased the time to exhaustion by 51% [132] and other per-cooling strategies such as the ingestion of cold fluids [114], the application of cooling garments [123,124,138], and the use of a neck cooling collar [117] induced an improvement in exercise performance ranging between 9 and 18%. In studies using menthol mouth rinse [130] or menthol applied to the face [127], performance was improved by 9% in trained athletes and by 18–21% in untrained participants.

Similarly, it has been noted that per-cooling interventions improve performance in time trial tests. As reported by Teunissen et al. [128], the application of wind cooling in a 15-km cycling time trial in the heat improved performance by 4%. Likewise, palm cooling enhanced a 30-km cycling time trial performance in the heat (32 °C) by 4 min (6.6%, ES = 1.54) [135]. In this work, a hand-cooling device that used a combination of negative pressure and a heat sink to increase the heat exchange between the circulating blood and the external environment was applied. As a result, cooler blood was delivered directly to the body core via venous return. Additionally, studies using neck cooling via a cooling collar showed a performance enhancements ranging between 6 and 13% in time trial protocols [116,117,118,120].

However, some other per-cooling strategies have been ineffective in enhancing performance. For instance, wearing a cooling vest during a 5-km running time trial in temperate conditions (25 °C) did not enhance performance [121]. Furthermore, despite a significant improvement in perceptual measures of exertion, thermal sensation and thermal comfort, menthol sprayed on a jersey in a 16.1-km cycling time trial in the heat did not improve performance time.

**Summary:**

➢ In summary, the majority of per-cooling methods have demonstrated significant improvements in endurance capacity and performance.
➢ The performance improvements from the per-cooling interventions tend to be larger in time to exhaustion tasks (9–51%) than in time trials (3–9%) [107]. These results may be explained by the fact that participants in time to exhaustion trials could not freely choose their self-paced intensity. Per-cooling may have larger beneficial effects on this type of exercise if athletes are allowed to choose their pace.

### 3.2. Per-Cooling and ‘Anaerobic’ Performance

During a session of repeated maximal sprints of 5 s, wearing an ice vest improved the ability of tetraplegic athletes to repeatedly perform sprints [139]. These athletes were able to improve their exercise capacity by completing eight additional sprints when wearing a cooling vest. Similarly, Sunderland et al. [140] evaluated the effect of neck cooling devices during repeated sprint exercises (5*6 s) before and after 45 min bouts of an intermittent football exercise (15 min*2). The authors reported a beneficial effect of neck cooling on the ability of athletes to repeat sprints and no changes in physiological responses. Additionally, cooling the torso and neck during exercise improved the athletes’ exercise capacity and performance in the heat [116,117,118,123]. Moreover, cooling the neck may be beneficial during intermittent activity in the heat, even when one’s core temperature exceeds 39.5 °C [141,142,143,144].

Furthermore, using an intermittent palm cooling device during a repeated leg press resistance exercise resulted in a higher power output [145,146]. It has been hypothesized that palm cooling allows a lower level of inhibition in the number of activated motor units, which results in a higher power output and number of repetitions [146].

LaFata et al. [147] investigated the effects of internal cooling by cold water ingestion on bench repetitions to fatigue, broad jumps and total time to exhaustion. The authors found no improvements in broad jump performance, no improvements in the TTE performances tests, and a decreased performance in the bench press. Such findings were explained by the relatively short duration of the exercises and a relatively small increase in the participants’ core temperature. Therefore, there was no need for cooling the body.

Similarly, head cooling by applying cold packs during brief (5s) and sustained maximal voluntary contractions (MVC) of the plantar flexors did not induce any beneficial effects on neuromuscular function [17].

**Summary:**

➢ The effects of cooling on ‘anaerobic’ exercise depend on the duration of the cooling method and its impact on decreasing the core and skin temperatures.
➢ The efficacy of per-cooling depends on the participant’s core temperature, and it has been hypothesized that a higher core temperature leads to a greater per-cooling impact on performance. Thus, the use of per-cooling should be recommended especially when the ambient temperature is high and/or the core temperature of the subject is expected to be elevated.

### 3.3. Per-Cooling and Cognitive Performance

Cognitive performance improves with mild heat strain but is impaired in individuals with a high degree of hyperthermia [148]. Cooling strategies seem to enhance cognitive performance, which is impaired by heat strain [92]. It has been reported that external cooling serves to alleviate the alterations induced by hyperthermia, such as thermoregulatory responses, and may reduce the difficulties the brain faces in dealing with accumulating stressors (i.e., cognitive demand and increasing heat strain), thereby allowing an increase in attentional resources to enhance cognitive functioning [92].

It has been acknowledged that maintaining a modest increase in core temperature and decreasing skin temperature improve cognitive performance in athletes performing in hot conditions. Therefore, external cooling, such as using an ice vest and cooled packs, may promote skin cooling without hindering the elevation of the core temperature [149]. However, internal cooling (ingestion of an ice slurry, cold water, etc.) may be less advisable because it could inhibit the initiation of hyperthermia and prevent cognitive function from reaching an optimal level.

In individuals with hyperthermia, the brain temperature may become higher than the core temperature [150], and cognitive effort cannot be sustained over time [90,151]. To address this problem and protect the brain, cooling methods such as head and neck cooling [120,148] may be of primary importance for sport performance and/or medical assistance.

Furthermore, head cooling seems to prevent the hyperthermia-related debilitative effects on short-term memory [14], and it also has a protective effect on cognitive performance during stressful conditions [152]. In Racinais’s study [17], cooling the head by applying cool packs was shown to slow the hyperthermia-induced changes in the working memory capacity, and it was discussed that the decrement in memory capacity could be due to frontal lobe activity. Simmons et al. [86] reported that high skin and core temperatures also induce changes in cognitive performance by a faster reaction time and a loss of accuracy (faster responses but more mistakes). However, the authors did not observe any beneficial effects of head cooling and expressed that the forehead, which is theoretically the most effective area in reducing thermoregulatory responses to a hot environment, was not directly cooled [86].

It is important to highlight that cognitive disturbances induced by hyperthermia are dependent on task complexity [153]. In this line, neck cooling has been found to be beneficial for improving performance in complex cognitive tasks (search and memory functions) [120]. Neck cooling may slow the perceptual high temperature experienced and may stop the progression of the ‘cognitive load’, thereby allowing a partial restoration of cognitive resources and enhanced performance in complex cognitive tasks in the heat [120]. However, further research are needed as several studies performed in a hot environment investigating the effect of cooling on cognitive performances using either head cooling [14,86] or neck cooling [120,154] revealed either no effect [86,154] or even an improvement of more complex cognitive tasks [120].

**Summary:**

➢ Cooling interventions such as head cooling and external cooling seem to restore cognitive resources disturbed by hyperthermia, increase performance in cognitive tasks and lower the perceived load of high temperature.

## 4. The Effects of Cooling Different Areas of the Body on Performance

The efficacy of the cooling method depends on the site at which cooling is applied and may be linked to tissue perfusion (e.g., the limited effects of cold water and slurry ingestion are due to the reduced splanchnic blood flow during exercise in the heat [155]). Hence, continuous cooling over an area with high tissue perfusion seems to be an ideal method for attenuating increases in body temperature [129].

### 4.1. Cooling a Large Area of the Body (e.g., Whole Body Cooling)

Vigorous cooling techniques that cover a large part of the body appear to be the most effective for improving performance [156]. For example, when cooling the torso and/or lower limbs, a large surface area is refreshed, and there are possibilities for effective heat exchange [26]. Therefore, a larger surface area covered by the cooling method leads to a higher rate of heat exchange [157].

Previous data showed that compared with cooling a small part of the body, cooling a large surface area during physical exercise in an environment with a hot ambient temperature leads to an increased work capacity and a greater suppression of physiological load [158]. Therefore, studies have shown that using whole body water immersion lowers physiological and thermal loads [159,160] and improves exercise performance [161,162,163,164]. Furthermore, the cooling rate seemed to be related to the specific body surface that was immersed in cold water [165]. In this line of work, Choi et al. [166] suggested that the heat strain can be considerably eliminated by the combined use of a cooling vest, a scarf, and a brimmed hat, which leads to a total cooling area of 4.2% of the body surface area (BSA).

In addition, during exercise in the heat when most of the body surface area is cooled by circulating cool water [167] or by a thermally isolated environment [168], sweating is clearly reduced and body temperature increases are minimized. In addition, when a relatively large part of the BSA is cooled, such as the torso alone [169] or the combination of the upper arms, torso and thighs [170], a substantial reduction in heat strain takes place.

However, it has been shown that cooling 15% of the BSA resulted in a complete elimination of heat strain [167]. However, in some circumstances, using such a cooling strategy on a large area of the body is not feasible [171]. Hence, if cooling only a part of the body surface ensures an effective reduction in heat strain, cooling the whole body is not necessary.

**Summary:**

➢ When cooling covers a large surface area of the body, a great reduction in heat strain can be obtained due to heat exchange. However, cooling only a part of the body where high tissue perfusion exists may be sufficient for full heat strain dissipation.

### 4.2. Torso Cooling

Regarding the body region, the torso can easily be cooled using a cooling vest. Choi et al. [166] demonstrated that the vest (torso cooling) with a cooling area of only 3.3% of the BSA was effective in alleviating heat strain. The beneficial effects of chest cooling may be explained by the large vascularization in this area, which allows the cooling of substantial quantities of blood [172]. In addition, a previous study [173] tested the effects of chest cooling on performance in severe heat and showed an improvement in tolerance time, which increased from 35 to 120 min. Additionally, Shvartz tested the effects of cooling either the neck or the chest using a circulating cool water (8.3 °C) system with heat exchangers and found that cooling the chest is as efficient as cooling the neck [172].

Nevertheless, for some other authors, cooling the whole chest was more effective in alleviating heat strain than cooling the neck was [174,175]. This finding may be due to a higher sensitivity of the trunk to cold temperatures due to the presence of a higher density of cold receptors in the trunk [176]. Nonetheless, as observed by Toner et al. [177], cooling both the chest and arms may heighten the cooling rate and improve performance during upper body exercises to a greater extent than cooling the torso.

**Summary:**

➢ The chest area has a high sensitivity to cold temperatures, which promotes its role in minimizing heat strain. The cooling of both the chest and another part of the body (e.g., the arms) may be more advantageous to improve performance.

### 4.3. Head and Neck Cooling

It has been emphasized that the cooling of only a limited area of the body surface may be sufficient to obtain an optimal reduction in heat strain [117,178].

The head and neck regions are characterized by a greater density of cold-sensitive afferent thermal receptors than the oropharyngeal cavity is [179]. Therefore, head and neck “cooling” using menthol [116,119,127] resulted in a more efficient cooling effect than did cooling the oropharyngeal cavity [130]. Therefore, as mentioned by Cotter and Tyler [179], the head and face are sites of high alliesthesial thermo-sensitivity. Thus, cooling these areas leads to more enhanced thermoregulatory responses than does cooling other parts of the body [172]. In this line, Shvartz et al. [180] compared the cooling of the head and neck with the cooling of other areas of the body (torso, arms and thighs) using a circulating cool water system through a hood and a suit. In men working in hot conditions, cooling the neck and the head were more effective and efficient than cooling other parts of the body.

Despite the fact that the head and the neck comprise only 10% of the total body surface area [181], head and neck cooling was found to be as effective as cooling approximately 60% of the body surface area in alleviating heat strain [116,119,127,132,180,182,183] and thereby enhanced exercise performance in an efficient way [179].

In a recent meta-analysis, Chan et al. [184] compared hand cooling with cooling of both the head and the neck by using small cooling packs in the context of exercising in the heat and found no significant differences in the changes in the physiological or psychological variables that were studied. However, they found that head and neck cooling significantly and positively impacted human performance with a small effect size (6.8%, EFS = 0.36), whereas hand cooling produced a negligible effect (−5%, EFS = 0.13).

In regard to neck cooling, previous data recommended its use owing to practical considerations (the neck is more easily accessible than other areas of the body) as well as anatomical and perceptual specificities (e.g., the neck is in close proximity of the thermoregulatory centre and is a region of high alliesthesial thermo-sensitivity) [179]. Moreover, cooling the neck may be advantageous due to its proximity to large blood vessels (enabling heat removal) [172] and its generation of feelings of thermal comfort when the thermo-sensitive receptors are stimulated [185].

**Summary:**

➢ An optimal amount of heat removal can be obtained through head and neck cooling due to the density of cold-sensitive afferent thermal receptors in these areas.
➢ Despite the small surface area (10%) of the head and neck, cooling of the head and neck regions was found to be as effective as the cooling of approximately 60% of the body surface area.
➢ Head and neck cooling enhance performance and perceived sensations in hot conditions.

### 4.4. Face Cooling

When compared with other areas of the body, the face tended to show stronger discomfort during heat exposure, and facial cooling was found to be comfortable [186,187]. The face region is more sensitive to cooling than other parts of the body due to its important sudomotor and alliesthesial thermosensitivity to cooling. Thus, cooling the face resulted in a two-to five-fold more powerful suppression of sweating and thermal discomfort than did the cooling of a surface area of an equivalent size [179]. Hence, facial cooling can be used to optimally reduce thermal discomfort, as previously suggested [188,189,190].

**Summary:**

➢ The face area displayed a high sensitivity to cooling, resulting in a two- to five-fold more powerful suppression of sweating and thermal discomfort than the cooling of other surface areas of equivalent size.

### 4.5. Hand Cooling

During heat exposure, the blood vessels of the limb and extremities are dilated to promote heat dissipation [185]. Thus, the hands have a high potential to attenuate increases in body temperature and reduce thermal discomfort during heat exposure [135,191]. To this end, some devices that are able to increase the rate of heat exchange between the circulating blood in the hands and the external environment are currently available. Therefore, the cooled blood returns to the core of the body via venous return [134,135,136] and reduces the core temperature.

Hsu et al. [135] demonstrated that this type of cooling significantly lowered tympanic temperature, lactate concentration, and oxygen uptake during a submaximal steady rate exercise test (1-h cycling at 60% VO_2_ max) and reduced the time required to complete a 30-km cycling time-trial test. However, regarding thermal sensation or thermal comfort, it has been reported that the limb extremities are less sensitive to high temperatures than the torso and the face [179]. This finding was confirmed later by Nakamura’s study, which showed that when the whole body was exposed to heat, local hand cooling decreased local thermal comfort, while the changes in whole-body thermal comfort were not of large magnitudes [185].

**Summary:**

➢ Although hands have a weak sensitivity to cooling, they can be deeply involved in the removal of heat and thus can reduce thermal strain.

In summary, based on the studies cited in this review, we can infer that the head and neck regions provided the greatest amount of heat removal, followed by cooling the torso and then cooling of other parts of the body (legs, hands, etc.) [192]. Therefore, it seems worth noting that regional cooling is effective for obtaining thermal comfort at a relatively low cost using compact systems that are easy to carry.

It is plausible that the efficacy of cooling depends on the area of the body that is cooled. However, the efficacy of cooling also depends on which muscle groups are activated and the types of exercises being performed. For instance, Young et al. [171] demonstrated that cooling of the arms during upper body exercises provided no thermoregulatory advantages, although cooling of the thigh surfaces during lower body exercises did provide an advantage. The higher ability of thighs to induce vasomotor adjustments may enable increased conductive cooling in the thighs compared with the arms.

## 5. The Underlying Physiological Mechanism of Per-Cooling

The combination of exercise and high environmental temperatures creates stress on the human body, which is countered by physiological responses [193]. It has been reviewed that heat-mediated fatigue can be induced by cardiovascular, thermoregulatory, cerebral and psychophysiological alterations [55,61,194]. It is likely that the use of per-cooling may serve to attenuate these factors. Notably, per-cooling has been shown to be more effective than pre-cooling in providing thermoregulatory benefits and reducing cardiovascular strain [195,196]. It has also been suggested that other mechanisms can also be responsible for the beneficial effects of per-cooling [194,197].

### 5.1. Thermoregulation Mechanisms

In hot environmental conditions, the production of heat in exercised muscles reaches a relatively high level. Therefore, the maintenance of a heat balance requires the involvement of dissipating mechanisms such as vasodilation and sweating [198,199]. To attenuate the increases in temperature and enhance heat loss, cooling strategies are employed during exercise. For instance, the study of Webborn et al. [139] showed a smaller increase in core temperature when an ice vest was applied that perhaps secluded the torso from heat gain. Some other studies confirm these findings [117,123]. Indeed, per-cooling may be an efficient means to attenuate the rise in core temperature during heat exposure, prevent a further degradation in exercise performance [27] and even provide performance enhancements [26,117]. Similarly, Burdon et al. [200] reported a beneficial effect of ingesting cold fluid (4 °C, 185 mL) during 90 min of cycling at 62% VO_2_ max at 30 °C on mean skin temperature. A recent meta-analysis also showed that per-cooling induced a decrease in skin temperature of 0.8 °C accompanied by an attenuation of the increase in core temperature and an improved exercise capacity [27]. Furthermore, per-cooling does not lower the sweat rate during exercise [105,106,196], and such a feature may lead to positive effects on heat loss [201].

Nonetheless, it is important to highlight that severe cooling of the periphery may significantly decrease the intramuscular temperature, which is known to slow enzymatic processes and nerve conduction, in turn reducing the rate of force generation and muscular endurance [202].

### 5.2. Cardiovascular Mechanisms

Hyperthermia induced by endurance exercises is characterized by an increased metabolic [203] and cardiovascular strain [57]. To reduce the thermal strain caused by hyperthermia, there is a need to accelerate the skin blood flow to facilitate heat loss, which possibly reduces the cardiac filling and increases the cardiovascular strain [204]. Therefore, cooling the skin by using per-cooling may attenuate cardiovascular strain [199] due to a reduced heart rate [205,206] and decelerated cutaneous circulation [207]. Cooling the skin with cooling garments significantly lowered the heart rate [196,208], elicited an increased preload and cardiac filling via a cold-induced cutaneous vasoconstriction and allowed a sustained cardiac output [209,210]. The underlying mechanism seems to involve the activation of baroreceptors when the blood at the skin level is redirected to the core level and the stimulation of the parasympathetic system. Subsequently, these changes resulted in a reduction in cardiovascular strain. Other studies confirmed this finding by using various cooling interventions during exercise, such as fanning [128,211], the application of an ice vest [123,212] and the ingestion of cold fluid [195,213]. Conversely, Ruddock’s meta-analysis [129] showed no beneficial effects on heart rate, confirming improved endurance performance. Although the greatest improvement in performance was observed when both the core temperature and the heart rate were reduced by cooling [123], a possible improvement was also observed in the absence of a reduction in heart rate or core temperature [116,117,118,132].

### 5.3. Central Nervous System Adjustments

In the heat, central fatigue may be induced by a reduction in cerebral blood flow [214], inhibition of motor activations [215], and a low serotonin/dopamine ratio in the hypothalamus [216]. Cooling may mitigate these effects. In fact, several researchers have alluded to the effect of per-cooling on the central nervous system mechanisms [105,116,130,132]. Dopamine, serotonin and noradrenaline seem to be important regulators of the central nervous system in hot environmental conditions [217]. Prolactin has been proposed as a peripheral marker of central fatigue in hot environments, and it has been highlighted that its release is inhibited by dopaminergic activity and stimulated by serotonergic activity [218]. In hyperthermic conditions, blood prolactin concentration was significantly reduced by using facial cooling during exercise [132,219,220]. This finding was recently confirmed during a running time trial by both spraying facial water and rinsing with a menthol mouth rinse [106]. Likewise, neck cooling during repeated sprint exercises led to a lower prolactin concentration during the later stage of the exercise session [140]. Nevertheless, studies on neck cooling during prolonged exercise have not shown any effects of these factors on the prolactin concentration in blood [116,118].

In summary, most of the studies cited above showed a reduction in the concentration of prolactin when per-cooling was used, which might suggest that central fatigue was lower with per-cooling.

### 5.4. Perceptual Mechanisms

Noticeably, per-cooling seems to improve perceptual measures such as thermal perception and the rating of perceived exertion (RPE), thereby inducing better self-selected intensities during time trials [129].

Studies using the application of menthol [130,131] during exercise showed improvements in thermal perception via the stimulation of cold receptors located in the skin when the topical gel was used [127] and in the oropharyngeal cavity when the mouth rinse was used [130]. Enhancements in thermal perception may generate a cool feeling in athletes [127,221], which may reduce thermal strain and improve performance [194,197]. Neck cooling during heat exposure generated feelings of thermal comfort at rest [185] and during exercise [116,120]. Hence, increasing perceptual comfort may allow individuals to conduct work for longer periods.

During endurance exercise [123,130,213], per-cooling appears to be beneficial in reducing perceived exertion and may permit an increase in self-selected work output and improved performance [12]. In addition, the beneficial effects of per-cooling during high intensity exercises may be linked to a lower amount of micro-damage in the muscle tissue; consequently, with a lower amount of muscle fatigue, the exercise can be sustained for a longer time [139]. Nevertheless, athletes must be aware of a false sense of lowered thermal perception which may lead them to prolong their activity and increased their risk for heat-related illnesses.

## 6. Conclusions

We have shown in this narrative review that using cooling devices during physical exercise may induce some positive effects on physical and cognitive performance. These positive effects are of main importance in the context of sports (e.g., Tokyo Olympic Games in 2020 that are going to be organized during a very hot and humid season, Football World Championship in 2022 in Qatar with a hot and dry environment) and in the context of global warming. It is known that health problems may develop during heat wave periods, especially in people who are frequently exposed to environmental stressors (e.g., workers in building companies, miners, farmers, …), weak and frail individuals (e.g., older people, patients with chronic diseases), and individuals who have difficulties with thermoregulation (pregnant females, babies, children, elderly individuals).

It is clear that many research studies will be conducted in this area in the near future to develop cooling devices. The design of the devices should make them practical, easy to use, effective, comfortable, light enough, easy to transport and relatively cheap. The selection of a device will depend on the needs of an individual, and a large panel of devices with different characteristics should be made available. Some users will require strong and long-lasting cooling power, and others will require comfort and ease of movement when wearing the device. Additionally, depending on the characteristics of the individuals, the devices may be different. For instance, depending on his or her thermoregulating capacity, his or her need for cooling, the ambient environment, and the task to be performed, an individual may choose to use a cooling vest instead of a neck collar or a cooling cloth made with cooling materials. A deeper understanding of human physiology will also be needed, especially of the physiology of athletes, females, children, and older individuals. For instance, there are very few studies available in individuals with very high aerobic fitness (e.g., athletes). It has been shown that when these individuals are exposed to heat, their skin temperature raises at a relatively slow rate as they promptly sweat. Therefore, the use of a cooling device at the beginning of a physical activity may not be advantageous, and if a cooling device is used, it may delay the cooling process. Males and females also behave differently in terms of thermoregulation, and depending on the sex of an individual, the characteristics of the devices may differ. More information is needed for female subjects in general, as the majority of the studies have been conducted in males. There is a clear lack of data concerning female athletes.
